# HS-5 and HS-27A Stromal Cell Lines to Study Bone Marrow Mesenchymal Stromal Cell-Mediated Support to Cancer Development

**DOI:** 10.3389/fcell.2020.584232

**Published:** 2020-11-05

**Authors:** Annalisa Adamo, Pietro Delfino, Alessandro Gatti, Alice Bonato, Paul Takam Kamga, Riccardo Bazzoni, Stefano Ugel, Angela Mercuri, Simone Caligola, Mauro Krampera

**Affiliations:** ^1^Stem Cell Research Laboratory, Section of Hematology, Department of Medicine, University of Verona, Verona, Italy; ^2^Department of Medicine, Section of Immunology, University of Verona, Verona, Italy; ^3^Department of Diagnostic and Public Health, University of Verona, Verona, Italy; ^4^EA4340-BCOH, Biomarker in Cancerology and Onco-Haematology, UVSQ, Université Paris Saclay, Boulogne-Billancourt, France

**Keywords:** mesenchymal stromal cells, stromal cell lines, tumor biology, immunomodulation, tumor escape

## Abstract

In this study, we compared the overall gene and pathway expression profiles of HS-5 and HS-27A stromal cell lines with those of primary bone marrow MSCs to verify if they can be considered a reliable alternative tool for evaluating the contribution of MSCs in tumor development and immunomodulation. Indeed, due to their easier manipulation *in vitro* as compared to primary MSC cultures, several published studies took advantage of stromal cell lines to assess the biological mechanisms mediated by stromal cells in influencing tumor biology and immune responses. However, the process carried out to obtain immortalized cell lines could profoundly alter gene expression profile, and consequently their biological characteristics, leading to debatable results. Here, we evaluated the still undisclosed similarities and differences between HS-5, HS-27A cell lines and primary bone marrow MSCs in the context of tumor development and immunomodulation. Furthermore, we assessed by standardized immunological assays the capability of the cell lines to reproduce the general mechanisms of MSC immunoregulation. We found that only HS-5 cell line could be suitable to reproduce not only the MSC capacity to influence tumor biology, but also to evaluate the molecular mechanisms underlying tumor immune escape mediated by stroma cells. However, HS-5 pre-treatment with inflammatory cytokines, that normally enhances the immunosuppressive activity of primary MSCs, did not reproduce the same MSCs behavior, highlighting the necessity to accurately set up *in vitro* assays when HS-5 cell line is used instead of its primary counterpart.

## Introduction

Mesenchymal stromal cells (MSCs) are a heterogeneous cell population representing the progenitors of stromal tissues and containing multipotent cells capable of differentiating *in vitro* and *in vivo* into mesodermal tissues, such as osteoblasts, chondrocytes, and adipocytes (Campagnoli et al., 2001; Im et al., 2005; da Silva Meirelles et al., 2006). In addition, MSCs are provided with immunomodulatory functions that are elicited by the presence of an inflammatory microenvironment. This phenomenon, called “MSCs licensing,” induces MSCs to become strongly inhibitory towards different immune effector cells (IECs) of both innate immunity, such as neutrophils, monocytes and natural killer (NK) cells, and adaptive immunity, such as T cells, B cells and dendritic cells (Krampera, 2011; Di Trapani et al., 2016). MSC-mediated immunosuppression has been confirmed by several preclinical and clinical studies related to a large spectrum of inflammatory and autoimmune diseases, such as Graft-versus-Host Disease, Crohn’s disease, sepsis, colitis, acute kidney injury, autoimmune encephalomyelitis, and other disorders (García-Olmo et al., 2005; Le Blanc et al., 2008; Gonzalez-Rey et al., 2009; Patel and Genovese, 2011; Ciccocioppo et al., 2012; Ciccocioppo and Corazza, 2016; Dal Collo et al., 2020). The well-known molecular mechanisms involved in MSC-mediated immunosuppression are represented by the up-regulation of several immunosuppressive molecules, including IDO1 and PD-L1 (Krampera, 2011; Di Trapani et al., 2016). Moreover, the role of FasL expression on MSCs cell surface has been recently reported to induce Fas-mediated T cell apoptosis (Akiyama et al., 2012).

In the last years, MSCs have been further recognized as crucial facilitators of tumor development in the context of both solid and liquid cancers. Emerging data suggest that MSCs can promote different tumor processes, including malignant transformation, angiogenesis, metastasis formation, cancer cell survival and chemoresistance (Nwabo Kamdje et al., 2017; Ridge et al., 2017; Galland and Stamenkovic, 2020). Last, but not least, the immunosuppressive properties of MSCs play a crucial role in mediating the mechanisms of immune escape in the context of tumor (Galland and Stamenkovic, 2020). Therefore, MSCs can be recruited within the tumor environment and establish dynamic interactions with tumor cells and other cellular elements, including IECs, by paracrine or contact-mediated communication (Galland and Stamenkovic, 2020; Le Naour et al., 2020). On the other hand, MSCs can also influence tumor growth by endocrine signals through the release of bioactive factors, including extracellular vesicles (Adamo et al., 2019a,b).

Several recent studies have tried to characterize the molecular mechanisms underlying the interactions amongst IECs, cancer cells and MSCs (Whiteside, 2018; Adamo et al., 2019a; Wei et al., 2019). These efforts may allow to identify novel potential therapeutic targets not only in the field of inflammatory and autoimmune disease, but also in the context of solid tumors and hematological malignancies. Considering the heterogeneity of MSC populations and that they may be potentially difficult to source, the use of commercially available bone marrow-derived cell lines, such as HS-5 and HS-27A, may have some advantages to obtain reproducible disease models *in vitro*, with low variability of the results obtained in presence of stromal cells. Therefore, several research groups take advantage of such commercially available cell lines to study the mechanisms mediated by MSCs in influencing immune responses and tumor progression (Garrido et al., 2001; Windus et al., 2013; Bar-Natan et al., 2017). HS-5 is a fibroblast-like cell line secreting significant levels of granulocyte colony-stimulating factor (G-CSF), granulocyte-macrophage-CSF (GM-CSF), macrophage-CSF (M-CSF), Kit-ligand (KL), macrophage-inhibitory protein-1 alpha, interleukin-6 (IL-6), IL-8, and IL-11. Furthermore, HS-5 supports the proliferation of hematopoietic progenitor cells when co-cultured in serum-deprived media without exogenous factors (Roecklein and Torok-Storb, 1995). HS-27A cell line shows an epithelioid morphology with much larger cell size as compared to HS-5, poorly secreting growth factors and not supporting the proliferation of isolated hematopoietic progenitor cells in co-cultures. Similarly, HS-27A-derived conditioned medium fails to support the growth of myeloid colonies (Roecklein and Torok-Storb, 1995). Therefore, it is likely that HS-5 and HS-27A might represent functionally distinct components of the bone marrow stromal microenvironment (Roecklein and Torok-Storb, 1995). However, further and detailed comparison is still missing concerning the capability of such cell lines to reproduce typical functional properties of primary bone marrow MSCs, including immunoregulatory functions. Theoretically, the use of immortalized cell lines in experimental procedures might have a number of advantages to evaluate the molecular mechanisms underlying tumor immune escape, due to their easier manipulation *in vitro* as compared to primary cultures. On the other hand, it is necessary to assess carefully whether mesenchymal cell lines may accurately reproduce the physiological properties of primary MSCs, considering that the immortalisation process could profoundly alter gene expression profile, and consequently biological characteristics.

## Materials and Methods

### Datasets, Expression Profiles and Statistical Analysis

NCBI Gene Expression Omnibus (GEO) database was searched for datasets with publicly accessible datasets with the keywords MSC, HS-5 and HS-27A. We selected four microarray datasets, GSE9593 (Wagner et al., 2008), GSE10595 (Iwata et al., 2014a), GSE48302 (Paul et al., 2013) and GSE53199 (Iwata et al., 2014b) containing samples that were eligible for our analysis. The details of the datasets and samples used are reported in Table 1. The following procedure was employed to account for the batch effect differences across the datasets and make the expression profiles comparable. First, platform-specific normalized data were downloaded with the *GEOquery Bioconductor* R package (Davis and Meltzer, 2007); multiple probes mapping to the same gene were collapsed by mean values; each dataset was subsetted to the samples indicated in Table 1; each dataset was individually quantile normalized using the function *normalize.quantiles.use.target* from the *Bioconductor* package *preprocessCore*, using as target distribution the quantile normalization vector available at https://api.refine.bio/v1/qn_targets/homo_sapiens, prepared by the refine.bio project^1^; all the four datasets were then merged and the dataset batch effect was removed with the *removeBatchEffect* function from *limma* package (Ritchie et al., 2015). Gene sets collections were obtained from MSigDB database (Subramanian et al., 2005) and to obtain gene sets/pathways expression levels we employed the *GSVA Bioconductor* package (Hänzelmann et al., 2013) and the *gsva* function, applied to the merged expression matrix. GSVA scores were used to compare the pathways expression levels between cell lines and the *eBayes* function from *limma* was used to compute moderated t-statistics after linear model fitting. Statistical significance was set at FDR < 0.05 and all the *p*-values reported in the boxplots represent adjusted *p*-values. All statistical analyses were performed with *R* software environment version 3.6.2.

**TABLE 1:** Details of the datasets and samples used for the expression profiles comparison.

GEO ID	Technology	PMID	Samples Used	Platform ID	Samples ID
GSE9593	Microarray	18493317	MSC	HG-U133_Plus_2	GSM242651, GSM242652, GSM242653, GSM242666, GSM242667, GSM242668, GSM242669, GSM242672, GSM242673, GSM242674, GSM242675
GSE10595	Microarray	24131213	HS-5, HS-27A	HG-U133_Plus_2	GSM267077, GSM267078, GSM267081, GSM267082
GSE48302	Microarray	24090675	HS-5, HS-27A	Illumina HumanHT-12 V3.0	GSM1174437, GSM1174438, GSM1174439, GSM1174440
GSE53199	Microarray	25275584	HS5	Illumina HumanHT-12 V4.0	GSM1287201, GSM1287202

### Cell Cultures

Primary MSCs were isolated from BM aspirates of healthy donors under informed consent, as approved by Ethical Committee of Azienda Ospedaliera Universitaria Integrata Verona (N. 1828, May 12, 2010 “Institution of cell and tissue collection for biomedical research in Onco-Hematology”) and characterized as already described (Di Trapani et al., 2016; Adamo et al., 2019a). HS-5 and HS-27A human stromal cell lines were obtained from ATCC^®^ (ATCC^®^ CRL-11882^TM^ and ATCC^®^ CRL-2496^TM^, respectively). Both primary MSCs and cell lines were cultured in DMEM supplemented with 10% heat-inactivated fetal bovine serum (FBS), 1% penicillin-streptomycin, and 2% L-Glutamine (all from Sigma Aldrich). All experiments were performed between passages 2 and 7 of primary MSCs. Cells at 80% confluence were treated or not for 48 h with 10 ng/mL IFN-γ and 15 ng/mL TNF-α (R&D Systems) to induce inflammatory priming. PBMCs were isolated from human blood using Lymphoprep (Stem cells Technologies). B, T, and NK lymphocytes were isolated from PBMCs using immunomagnetic negative selection (Miltenyi Biotec) with at least 95% cell purity, as evaluated by flow cytometry. PBMCs were stimulated with 5 μg/ml of phytohemagglutinin (PHA) (Sigma-Aldrich) for 4 days in IMDM supplemented with 10% pooled human AB serum, 1% penicillin-streptomycin and 2% L-Glutamine (all from Sigma-Aldrich). T cells were activated with 0.5 μg/mL cross-linking anti-CD3 and anti-CD28 antibodies (Sanquin) for 6 days in RPMI supplemented with 10% human AB serum, 1% penicillin-streptomycin and 2% L-Glutamine (all from Sigma-Aldrich). B cells were activated with 5 μg/mL antihuman IgM+IgA+IgG (F(ab’)2, Jackson Immunoresearch), 50 IU/mL rhIL-2 (Novartis), 50 ng/mL polyhistidine-tagged CD40 ligand, 5 μg/mL anti-polyhistidine antibody (R&D Systems), and 0.5 μg/mL CpG ODNs (Invitrogen), in RPMI medium supplemented with 10% FBS, 1% penicillin-streptomycin and 2% L-Glutamine (all from Sigma-Aldrich). The identity of HS-5 and HS-27A was checked for the presence of mesenchymal markers. Cell suspension were stained using the antibody anti-human CD73-PE, CD90-PE, CD105-PE, CD14-PE, CD31-PE, CD34-PE, and CD45-PE, HLA-ABC-PE, HLA-DR-PE, Fas-FITC, FasL-PE (BD Bioscience). The inflammatory immunophenotype was established using the anti-human CD54-PE, CD106 PE, HLA-ABC-PE, HLA-DR-PE, CD274-PE monoclonal antibodies (BD Bioscience). Peripheral blood mononuclear cells (PBMCs) were characterized using the anti-human CD3-FITC, CD16/56-PE, CD45-PerCP, CD19-APC, CD4-APC-H7, and CD8-PECy7 monoclonal antibodies (BD Bioscience). All data were collected through flow cytometry (FACS Canto II, BD Bioscience) and analyzed with FlowJo software (TreeStar). The expression of MSC markers was analyzed on living cells by using TO-PRO^TM^-3 Iodide (Thermo Fisher) and normalized on FMO (fluorescence minus one) control. Osteogenic and adipogenic differentiative ability of primary MSC and γ-irradiated HS-5 and HS-27A cells (20 Gy – ^137^Cs as source of γ-radiation) was evaluated has already described (Di Trapani et al., 2016; Adamo et al., 2019a). Primary MSCs and the cell lines were negative for mycoplasma.

### Immunological Assays

Standardized assays were carried out to assess the inhibitory functions of primary MSCs and cell lines on different IECs, as previously described by our group (Di Trapani et al., 2016). Either primary MSCs or cell lines at resting and inflammatory-primed conditions were cultured in presence of activated PBMCs or purified T, B, NK cells previously stained with 5 μM carboxyfluorescein succinimidyl ester (CFSE) (Life Technologies). HS-5 and HS-27A were plated at 80% confluence. After cell adhesion, 2 × 10^5^ PBMCs, 2 × 10^5^ T cells, 2 × 10^4^ B cells, or 2 × 10^4^ NK cells were added. At the end of the co-culture, cells were harvested at the stained with mouse anti-human CD45-PerCP-Vio700 (Miltenyi Biotec), and TOPRO-3 Iodide (Life Technologies). The proliferation was assessed on viable TOPRO-3 negative and CD45 positive cells by FlowJo software (TreeStar) by using the CFSE Geometric Mean of proliferating cells. The same experimental procedures were carried out using γ-irradiated HS-5 and HS-27A cells (20 Gy – ^137^Cs as source of γ-radiation).

## Results

### HS-5 and HS-27A Cell Lines Display the Typical Markers Expression Profile of Primary MSCs, but With Different Intensity

**FIGURE 1:**
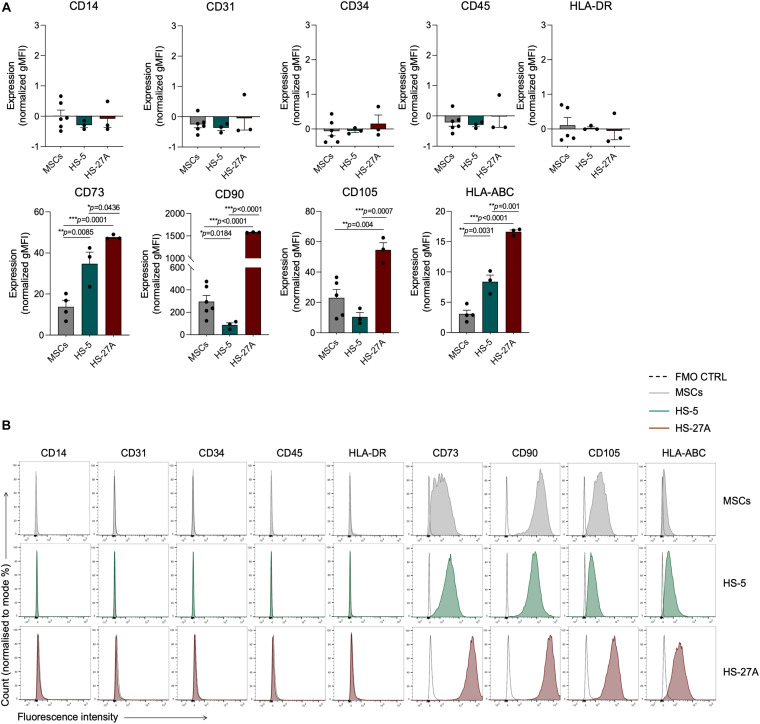
Markers expression profile of primary MSCs, HS-5, and HS-27A cell lines. **(A)** Flow cytometry immunophenotypic analysis of primary MSCs, HS-5, and HS-27A cell lines showing the expression profile of specific hematopoietic markers (CD14, CD31, CD34, CD45), mesenchymal stromal cell markers (CD73, CD90, CD105), and MHC class I and II molecules (HLA-ABC and HLA-DR, respectively). Data are represented as mean of gMFI (geometric mean fluorescence intensity) normalized on FMO (fluorescence minus one) control ± SEM. **(B)** Representative histograms showing the expression profile of hematopoietic markers (CD14, CD31, CD34, CD45), mesenchymal stromal cell markers (CD73, CD90, CD105), and MHC class I and II molecules (HLA-ABC and HLA-DR, respectively) on MSCs, HS-5 and HS-27A cell lines.

According to the minimal criteria for defining MSCs established by the International Society for Cellular and Gene Therapy (ISCT), primary bone marrow MSCs and HS-5 and HS-27A cell lines were positive for CD73, CD90, CD105, and HLA-ABC, with no expression of CD14, CD31, CD34, CD45, and HLA-DR surface molecules (Figures 1A,B). However, the expression intensity of the positive surface markers was significantly different among the different cell types. CD73, CD90, CD105, and HLA-ABC displayed a uniform expression on primary MSCs, regardless of the different healthy donors considered (Figure 1A). HS-5 showed a statistically significant higher expression of CD73 and HLA-ABC and a lower expression of CD90 compared to primary MSCs (Figures 1A,B). HS-27A displayed a significantly higher expression of all the positive surface markers compared to both primary MSCs and HS-5 (Figures 1A,B). Overall, these data confirm the preservation of the well-defined MSCs immunophenotypic profile in HS-5 and HS-27A cell lines. To further characterize stromal cell lines, we tested the ability of irradiated HS-5 and HS-27a to differentiate into osteoblasts and adipocytes. Both HS-5 and HS-27A were partially able to differentiate into osteoblasts, while they did not show any adipogenic differentiation properties (Supplementary Figure 1).

### HS-5 Cell Line Recapitulates the General Expression Pattern of Primary Bone Marrow MSCs

**FIGURE 2:**
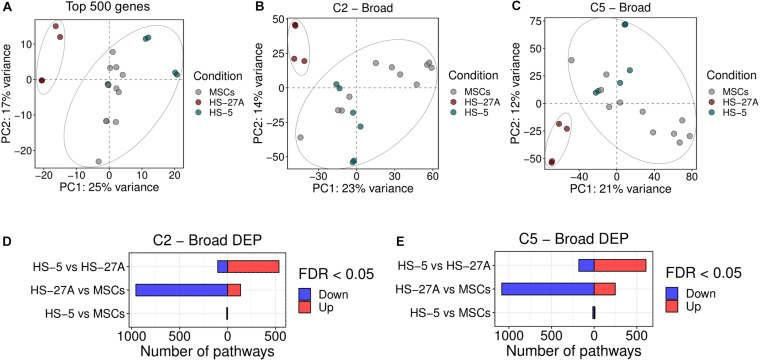
Overall gene expression profile of primary MSCs, HS-5 and HS-27A cell lines. **(A)** Score plot of the first two PCs calculated following the application of PCA on top 500 genes expressed by primary MSCs, HS-5, and HS-27 cell lines. **(B,C)** Score plot of the first two PCs following the application of PCA on DEP included in C2 **(B)** and C5 **(C)** gene sets collections between primary MSCs, HS-5 and HS-27 cell lines. **(D,E)** Number of significantly down- and up-regulated pathways included in C2 **(D)** and C5 **(E)** gene sets collections between primary MSCs, HS-5, and HS-27 cell lines. (*n* MSCs, HS-5, HS-27A = 11, 6, 4). PCs, principal components; PCA, principal component analysis; DEP, differentially expressed pathways.

We first compared the overall gene and pathway expression profile of primary bone marrow MSCs and HS-5 and HS-27A cell lines. As the comparison was based on a multi-datasets level, we applied a strategy to reduce the batch effect of the different datasets and we were able to control the datasets differences, as shown in the PCA and boxplot in Supplementary Figures 2A,B, respectively. From the PCA plot no clustering of the samples based on datasets was appreciable, while the boxplot of the normalized and quantile-transformed expression values outlined no evident differences across datasets. Indeed, exploring the variability of the cell lines based on PCA of the 500 top variable genes, we observed a clustering of the HS-5 sample cluster closer to MSCs, while HS-27A was confined and distinct from the other two cell types (Figure 2A). To further compare the overall differences between primary MSCs and cell lines, we took advantage of Gene Set Variation Analysis (GSVA) by exploring the Molecular Signatures Database (MSigDB), a collection of annotated gene sets^2^. For the initial evaluation of potential differences or similarities between primary MSCs and cell lines, we considered two general MSigDB gene set collections covering a good portion of the human cellular and biological pathways, i.e., C2 and C5 collections. C2 collection includes several gene sets deriving from various sources, i.e., online pathway databases and the biomedical literature. The C2 collection is divided into two sub-collections: Chemical and genetic perturbations (CGP) and Canonical pathways (CP). The majority of the CGP sets came from the biomedical literature, thus identifying different signatures of biological and clinical states, such as cancer metastasis, stem cell characteristics, etc. The CP sub-collection includes several pathway gene sets from commonly used online databases, including BioCarta, KEGG, Matrisome Project and others. C5 collection consists of gene sets derived from Gene Ontology (GO) annotations. Therefore, the C5 collection is based on GO terms, belonging to the three GO ontologies [molecular function (MF), cellular component (CC) or biological process (BP)], and their associations to human genes. Considering both C2 and C5 collection, the PCA analysis on differentially expressed pathways (DEP) clearly showed two distinct clusters. HS-5 cell line and primary MSCs clustered together, whereas HS-27A cell line represented a distinct group (Figures 2B,C). Surprisingly, HS-27A cell line displayed a substantial number of differentially expressed pathways compared to primary MSCs, considering both C2 and C5 collection (1091 and 1331, respectively) (Figures 2D,E). On the other hand, only 10 and 26 pathways included in C2 and C5 collections, respectively, resulted significantly modulated in HS-5 cell line as compared to primary MSCs (Figures 2D,E). Consequently, a high number of pathways were differentially expressed in the two cell lines (639 and 787 included in C2 and C5 collections, respectively) (Figures 2D,E). The lists of differentially expressed pathways in MSCs and cell lines by using C2 and C5 collections are available in Supplementary Tables 1, 2.

Taken together, these data indicate that HS-5, but not HS-27A, represents an immortalized cell line with a general expression pattern similar to the one observed in bone marrow-derived MSCs and, consequently, might be a reliable model to reproduce the biological properties mediated by MSCs.

### HS-5 Cell Line Recapitulates the Ability of Primary MSCs to Affect Tumor Biology

**FIGURE 3:**
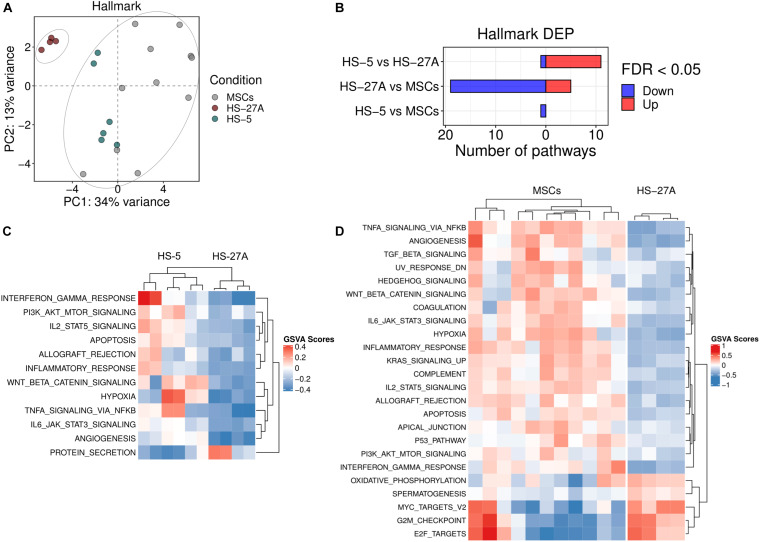
Tumor-affecting pathways expression on primary MSCs, HS-5 and HS-27A cell lines. **(A)** Score plot of the first two PCs following the application of PCA on DEP included in Hallmark gene sets collection between primary MSCs, HS-5, and HS-27 cell lines. **(B)** Number of significantly down- and up-regulated pathways included in Hallmark gene sets collection between primary MSCs, HS-5, and HS-27 cell lines. **(C)** Heatmap summarizing significantly DEP between HS-5 and HS-27 cell lines. **(D)** Heatmap summarizing significantly DEP between primary MSCs and HS-27 cell lines. (*n* MSCs, HS-5, HS-27A = 11, 6, 4). PCs, principal components; PCA, principal component analysis; DEP, differentially expressed pathways.

In the last years, MSCs have been extensively recognized as crucial players during the processes of tumor development in the context of both solid and liquid cancers. Emerging data suggest that MSCs can promote malignant transformation, angiogenesis, metastasis formation, cancer cell survival and chemoresistance (Ridge et al., 2017; Adamo et al., 2019b). Several reports took advantage of immortalized HS-5 and HS-27A cell lines to characterize such properties and to discover the molecular mechanisms underlying the dynamic interactions between MSCs and cancer cells. In order evaluate the reliability of using HS-5 and HS-27A as an alternative tool for the characterisation of primary MSC regulatory properties in the context tumor processes, we compared primary MSCs and immortalized cell lines as far as the expression profile of different available gene sets involved in tumor biology is concerned. In detail, we took advantage of Hallmark collection in MSigDB, which includes 50 gene sets. Among these, several signatures have been reported as crucial pathways responsible for the pro-tumor activity mediated by MSCs. PCA analysis on all the Hallmark gene sets clearly showed two distinct clusters related to primary MSCs and cell lines, as previously shown considering the C2 and C5 general dataset collections. HS-5 cell line and primary MSCs clustered together, whereas HS-27A cell line represented a distinct group (Figure 3A). Twenty-four pathways were differentially expressed in HS-27A as compared to primary MSCs. Among these, 19 resulted down-regulated and 5 up-regulated (Figure 3B). Several pathways that have been reported to be involved in MSC-dependent pro-tumor activity displayed a strong up-regulation in primary MSCs compared to HS-27A cell line, including “angiogenesis,” “Wnt/β catenin signaling,” “KRAS signaling,” “PI3K-AKT-mTOR signaling,” and many others (Wang et al., 2015; El-Badawy et al., 2017; Poggi et al., 2018; Adamo et al., 2019a; Figure 3D). Conversely, HS-5 cell line and primary MSCs displayed a similar pathway expression profile. Only the “Protein secretion” gene set was significantly down-modulated in HS-5 compared to primary MSCs (Figure 3B). As expected, the comparison between HS-5 and HS-27A cell lines revealed 12 DEP (Figures 3B,C). Overall, these data suggest that HS-5 is a more appropriate model to reproduce the typical MSC expression pattern responsible for the pro-tumor activity. Therefore, HS-5 cell line could be a reliable alternative to primary MSCs to deeply characterize the molecular interactions between stromal and cancer cells. The list of differentially expressed pathways in MSCs and cell lines according to Hallmark collection is available in Supplementary Table 3. Considering the well-established properties of MSCs to promote angiogenic processes, we reported in Supplementary Figure 3 the expression of all genes included in “angiogenesis” pathway from Hallmark MSigDB (Molecular Signature Database) in MSCs and stromal cell lines. As expected, several genes involved in such pathway resulted equally expressed in MSCs and HS-5, suggesting a similar ability in promoting angiogenic processes.

### HS-5 Cell Line Recapitulates the Ability of Primary MSCs to Affect Immune Responses

**FIGURE 4:**
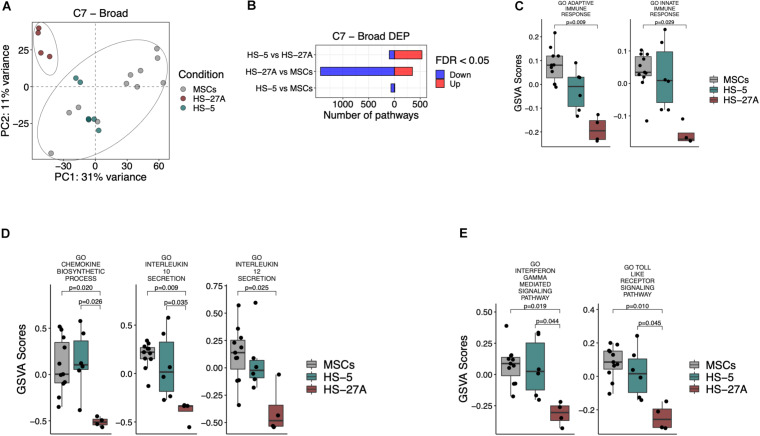
Immunological signatures on primary MSCs, HS-5, and HS-27A cell lines. **(A)** Score plot of the first two PCs following the application of PCA on DEP included in C7 immunological signatures collection between primary MSCs, HS-5 and HS-27 cell lines. **(B)** Number of significantly down- and up-regulated pathways included in C7 immunological signatures collection between primary MSCs, HS-5 and HS-27 cell lines. **(C–E)** GSVA scores related to the expression of selected immunological signatures responsible for the immunoregulatory activity mediated by MSCs on primary MSCs, HS-5 and HS-27 cell lines. (*n* MSCs, HS-5, HS-27A = 11, 6, 4). PCs, principal components; PCA, principal component analysis; DEP, differentially expressed pathways; GSVA, gene set variation analysis.

MSCs possess broad immunomodulatory functions affecting both innate and adaptive immune responses (Gao et al., 2016). In order to define the immunological expression profile of stromal cell lines in comparison to primary MSCs, we took advantage of C7 immunological signature collection in MSigDB, consisting of several gene sets involved in the regulation of the immune system. PCA analysis on all the immunological signatures in primary MSCs and cell lines confirmed the presence of two distinct groups. HS-5 cell line clustered within the group of primary MSCs, whereas HS-27A cell line represented a distinct group (Figure 4A). A higher number of immunological signatures resulted significantly different in HS-27A, rather than in HS-5, in the comparison with primary MSCs, i.e., 1785 and 74 DEP, respectively (Figure 4B). Moreover, the GO gene sets, including a wide list of genes implicated in the regulation of both adaptive and innate immune responses, resulted significantly up-regulated in primary MSCs as compared to HS-27A cell line, thus confirming that HS-5 is suitable to reproduce the immunological properties of primary MSCs (Figure 4C). To further compare primary MSCs and immortalized cell lines in terms of immunological properties, we analyzed the expression profile of the well-established GO immunological signatures responsible for the immunosuppressive activity mediated by MSCs. The synthesis and subsequent release of chemokines and cytokines by MSCs play a crucial role in regulating immune responses. Here, we showed that the processes involved in the biosynthesis of chemokines were strongly up-regulated in primary MSCs and HS-5 as compared to HS-27A (Figure 4D). In particular, the release of IL-10 and IL-12 positively correlated with MSCs suppressive effects (De Miguel et al., 2012; Kyurkchiev et al., 2014; Nakajima et al., 2017). As expected, the GO immunological signatures related to the release of IL-10 were up-regulated in both primary MSCs and HS-5 compared to HS-27A (Figure 4D). In addition, the GO immunological signatures related to the release of IL-12 was higher in primary MSCs compared to HS-27A, whereas we did not detect any difference between MSCs and HS-5 cell line (Figure 4D). Furthermore, we also investigated the expression of two pathways normally overexpressed during the immunosuppression mediated by MSCs. MSC inflammatory priming with IFN-γ enhances the immunosuppressive pathways responsible for the inhibition of different IECs (Krampera et al., 2006; Carvalho et al., 2019). Therefore, the IFN-γ-mediated signaling pathway can be considered as an essential signature of MSC-mediated immunosuppression. Such pathway was significantly enhanced in both primary MSCs and HS-5 compared to HS-27A (Figure 4E). The same trend was observed for “Toll-like receptor (TLR) signaling pathways” (Figure 4E), an additional biological system that may increase the immunosuppressive phenotype of MSCs (Najar et al., 2017; Shirjang et al., 2017). Overall, these data indicate that HS-5 is a more appropriate cell line to reproduce the immunological expression patterns responsible for the immunosuppressive activity of MSCs. The list of differentially expressed pathways in MSCs and cell lines according to C7 immunological signatures is available in Supplementary Table 4.

### Immunological Characterisation of HS-5 and HS-27A Cell Lines

**FIGURE 5:**
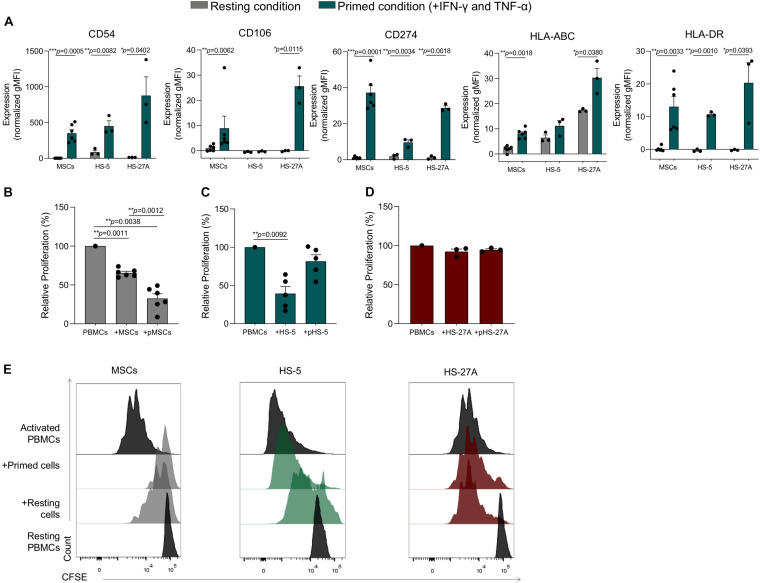
Immunological characterization of HS-5 and HS-27A cell lines. **(A)** FACS immunophenotypic analysis of primary MSCs, HS-5, and HS-27A cell lines showing the expression profile of CD54, CD106, CD274, HLA-ABC, and HLA-DR in resting or primed condition. Data are represented as mean of gMFI (geometric mean fluorescence intensity) normalized on FMO (fluorescence minus one) control ± SEM. **(B–D)** Relative PBMCs proliferation following 4 days of co-culture with resting or primed MSCs, HS-5 or HS-27A, respectively. PBMCs proliferation was calculated on living CD45^+^ cells according to CFSE dilution method by measuring CFSE gMFI and normalized on activated PBMCs cultured in absence of stromal cells. Data are represented as mean ± SEM. **(E)** Representative proliferation peaks of living CFSE^+^CD45^+^ PBMCs following the co-culture with resting or primed MSCs (gray), HS-5 (green), and HS-27A (dark red).

In order to validate our meta-analysis, we applied standardized assays to evaluate the immunological properties of immortalized cell lines. As previously reported by our group, the presence of inflammatory cytokines makes primary MSCs acquire an inflammatory phenotype characterized by increased expression of CD54 (I-CAM), CD106 (V-CAM), HLA-ABC, and HLA-DR (MHC-II), and CD274 (PD-L1) (Dal Collo et al., 2020). Furthermore, the inflammatory microenvironment induces a strong inhibitory effect on primary MSCs (primed MSCs, pMSCs), leading to the inhibition of immune responses mediated by different IECs (Di Trapani et al., 2016). In order to evaluate the immunological activity of HS-5 and HS-27A, we first assessed their phenotype in presence or not of inflammatory cytokines. Both HS-5 and HS-27A cell lines were capable of acquiring the typical phenotype of activated MSCs, except for the expression of CD106 on HS-5 that resulted absent both at resting and primed condition (Figure 5A). We also evaluated the expression of Fas and FasL in primary MSCs and stromal cell lines both in resting and primed condition (Supplementary Figures 4A,B). The expression of FasL by murine MSCs has been recently reported to be involved in Fas-mediated T cell apoptosis (Akiyama et al., 2012). In our cell models we observed a higher expression of FasL in stromal cell lines compared to primary MSCs (Supplementary Figure 4A) but the presence of inflammatory cytokines did not induce an up-regulation of the protein on the cell surface (Supplementary Figure 4B). As already reported (Yang et al., 2016; Martínez-Peinado et al., 2018), MSCs were able to significantly inhibit the proliferation of activated PBMCs, with a more pronounced effect when MSCs were pre-treated with inflammatory cytokines (Figures 5B,E). As expected, resting HS-5 led to a significant reduction of PBMC proliferation, as observed for primary MSCs, thus confirming its capability to reproduce the immunosuppressive activity mediated by MSCs towards PBMCs at resting conditions. However, the pre-treatment with inflammatory cytokines (pHS-5) did not affect PBMC proliferation (Figures 5C,E). HS-27A did not show any effect on PBMCs proliferation either at resting or primed conditions (Figures 5D,E). We observed similar results when HS-5 and HS-27A were γ-irradiated before the co-culture to prevent cell proliferation, and the immunosuppressive effect mediated by resting HS-5 was not observed any longer (Supplementary Figures 5A,B). Considering these data, we excluded the intrinsic ability of the two cell lines to induce the proliferation of resting PBMCs. HS-5 and HS-27A as well as primary MSCs were not able to activate resting PBMCs (Supplementary Figure 5C). Taken together, our data confirm a higher similarity of HS-5 to primary MSCs in terms of immunological activity.

### HS-5 Cell Line Reproduces the MSC Immunosuppressive Activity on Activated T, B, and NK Cells at Resting Conditions

**FIGURE 6:**
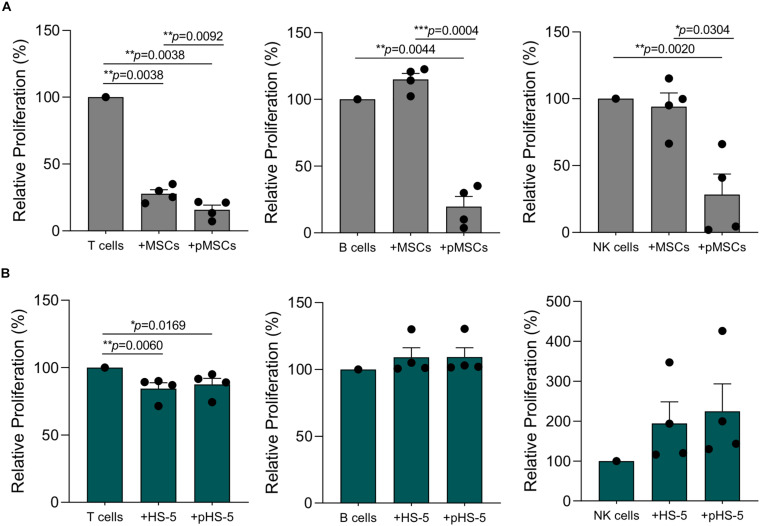
HS-5 cell line immunosuppressive activity on T, B, and NK proliferation in resting and primed condition. **(A,B)** Relative T, B, and NK proliferation following the co-culture with resting or primed MSCs (gray) and HS-5 (green), respectively. Cell proliferation was calculated on living CD45^+^ cells according to CFSE dilution method by measuring CFSE gMFI and normalized on activated IECs cultured in absence of stromal cells. Data are represented as mean ± SEM.

We further investigated HS-5 cell line immunological properties towards purified T, B, and NK cells by using standardized immunological assays. As already published by our group (Di Trapani et al., 2016), resting MSCs displayed a more significant suppressive effect on T cells as compared to other lymphocyte subsets (Figure 6A). These differences were partially related to the level of inflammatory cytokines released by activated IECs, which promoted the enhancement of MSC licensing. Accordingly, B and NK cell division was not inhibited by resting MSCs, due to their inability to make them acquire significant immunosuppressive activity (Figure 6A). Following IFN-γ and TNF-α pre-treatment, MSCs dramatically lowered T, B and NK cell proliferation by more than 80% (Figure 6A). As observed for primary MSCs, the co-culture with resting HS-5 induced a significant reduction of T cell proliferation, whereas we did not observe any effect on B and NK cell proliferation (Figure 6B). Conversely, the treatment of HS-5 cell line with inflammatory cytokines neither increased its immunosuppressive activity on T cell proliferation nor induced cell proliferation arrest of both B and NK cells (Figure 6B). As previously reported in the experimental setting of PBMCs, we did not observe any intrinsic ability of both HS-5 and primary MSCs to promote resting T, B, and NK cell proliferation (data not shown). Taken together, these data showed the capability of HS-5 cell line to reproduce the typical inhibitory effect of MSCs on T cell proliferation. However, the presence of inflammatory cytokines was not able to further enhance this phenomenon by using standardized immunological assay set up with primary MSCs.

## Discussion

The therapeutic potential of MSCs has been increasingly studied in the field of inflammatory and autoimmune diseases due to the ability of these cells to strongly suppress the immune responses. The well-established MSC immunomodulatory functions can be ascribed to their dynamic interactions with IECs mediating both adaptive and innate immune responses, through cell-to-cell contact and paracrine activity via soluble factors and extracellular vesicle release (Adamo et al., 2019a; Li et al., 2019; Zhou et al., 2019). The inflammatory microenvironment dramatically increases MSC immunosuppressive activity by influencing such interactions both *in vitro* and *in vivo* (Le Blanc et al., 2004; García-Olmo et al., 2005; Ciccocioppo et al., 2012; Di Trapani et al., 2016). MSC capability of affecting the immune responses plays a crucial role not only in the field of inflammatory disorders, but also in the context of tumors (Galland and Stamenkovic, 2020). In fact, MSCs can establish direct and indirect dynamic interactions with immune cells and favor the complex mechanisms of immune evasion. Furthermore, MSCs can influence a variety of tumor processes, directly promoting malignant transformation, angiogenesis, metastasis formation, cancer cell survival and chemoresistance (Nwabo Kamdje et al., 2017; Adamo et al., 2019b; Galland and Stamenkovic, 2020; Le Naour et al., 2020). Consequently, the characterisation of the molecular mechanisms underlying the interactions amongst IECs, cancer cells and MSCs may help to identify novel potential therapeutic targets. Immortalized cells are frequently used to describe the molecular mechanisms underlying the interactions between MSCs and various target cells. Some of these cell lines can support hematopoietic cell survival and proliferation, similarly to primary MSCs (Roecklein and Torok-Storb, 1995). However, little is known about the reproducibility and reliability of using mesenchymal cell lines in the field of immunomodulation and tumor biology. Here, we compared the overall expression profile of primary bone marrow MSCs with that of bone marrow-derived HS-5 and HS-27A cell lines. The aim of our study was to evaluate if HS-5 and HS-27A cell lines may represent standardized and reproducible cellular models to employ for the assessment of the molecular mechanisms underlying the reciprocal interactions of MSCs with IECs and cancer cells.

In our hands, only HS-5 cell line displayed a general expression pattern similar to the one observed in bone marrow-derived MSCs; instead, HS-27A did not. Consequently, HS-5 cell line could be a reliable model to reproduce the biological properties mediated by MSCs. This hypothesis was further and more strongly confirmed when we studied the pathways involved in tumor progression. We did not detect any differentially expressed pathway in primary MSCs and HS-5, except for the “Protein secretion” signature, thus suggesting that HS-5 cell line could help to characterize the molecular interactions between MSCs and cancer cells. Instead, HS-27A cell line could represent the negative control, as the majority of the gene signatures involved in the pro-tumor activity mediated by primary MSCs resulted down-modulated in this cell line, such as those regulating “angiogenesis,” “Wnt/β catenin signaling,” “KRAS signaling” and “PI3K-AKT-mTOR signaling” (Wang et al., 2015; El-Badawy et al., 2017; Poggi et al., 2018; Adamo et al., 2019a). The significant differences between HS-5 and HS-27A cell lines support the reliability of our method of comparison.

HS-5 cell line shared also the immunological signatures and the pathways responsible for the immunosuppressive activity of MSCs. In fact, we found that the processes involved in the biosynthesis of chemokines were strongly up-regulated in both primary MSCs and HS-5 cell line. As expected, the GO immunological signatures related to IL-10, IL-6, and IL-12 release were up-regulated in both primary MSCs and HS-5 cell line as compared to HS-27A. Similar findings were found as far as the expression of “IFN-γ-mediated signaling pathway” and “Toll-like receptor (TLR) signaling pathways” is concerned, two pathways strictly related to MSC immune regulatory effect. All these data were then confirmed by the functional assays we performed on activated PBMCs and purified IECs, although HS-5 pre-treatment with inflammatory cytokines, that normally enhances the immunosuppressive activity of primary MSCs, did not affect PBMC proliferation. This difference requires further investigation, because it could reflect either a different sensitivity of HS-5 cell line to inflammatory priming or a persistent status of intracellular activation.

Taken together, these data indicate that HS-5 cell line is suitable to reproduce not only the MSC capacity to influence tumor biology, but also to evaluate the molecular mechanisms underlying tumor immune escape mediated by stroma cells, with a number of advantages due to its easier manipulation *in vitro* as compared to primary MSC cultures. However, we strongly highlight and recommended to accurately set up the immunological assays when HS-5 cell line is used instead of its primary counterpart.

The pronounced differences between HS-5 and HS-27A reported in this work is further supported by Li et al. (2013) who showed that HS-27A, differentially from HS-5, can be co-injected in NSG mice with CD34^+^ cells isolated from myelodysplastic syndrome patients to promote the engraftment of clonal hematopoietic precursor. Furthermore, human CD34^+^ precursors harvested from bone marrow and spleen of primary murine recipients, when combined with HS-27A cells, were also engrafted successfully in secondary NSG recipients, showing the persistence of the original clonal characteristics (Li et al., 2013). The authors suggested that HS-27A stromal cells “traveled” in direct contact with hematopoietic precursors and enabled their propagation. An essential signal for engraftment appears to be CD146, which is prominently expressed on HS-27A cells compared to HS-5 (Li et al., 2013). Therefore, the higher levels of specific MSCs markers on HS-27A cell surface compared to HS-5 might probably be responsible for that capacity. In this light, HS-5 could represent a suitable model to study the immunoregulatory and tumor-promoting properties mediated by MSCs. On the other hand, HS-27A could be a reliable model to evaluate the role of MSCs in engraftment processes.

## Data Availability Statement

The original contributions presented in the study are included in the article/Supplementary Material, further inquiries can be directed to the corresponding authors.

## Ethics Statement

The studies involving human participants were reviewed and approved by Ethical Committee of Azienda Ospedaliera Universitaria Integrata Verona; N. 1828, May 12, 2010 “Institution of cell and tissue collection for biomedical research in Onco-Hematology”. The patients/participants provided their written informed consent to participate in this study.

## Author Contributions

AA designed, performed the laboratory work, and wrote the manuscript. PD performed statistical and gene expression profile analyses and wrote the manuscript. AG, AB, AM, PT, RB, and SC performed the laboratory work and contributed to manuscript writing. SU critically revised the manuscript. MK coordinated the research plan, wrote and approved the final version of the manuscript. All authors contributed to the article and approved the submitted version.

## Conflict of Interest

The authors declare that the research was conducted in the absence of any commercial or financial relationships that could be construed as a potential conflict of interest.

## Footnotes

1 https://www.refine.bio

2 https://www.gsea-msigdb.org

## Supplementary Material

Supplementary Figure 1 Osteoblasts and adipocytes differentiation of primary MSCs and irradiated stromal cell lines HS-5 and HS-27A.

Supplementary Figure 2 Overall gene expression profile of primary MSCs, HS-5, and HS-27A cell lines following batch effect normalization. **(A)** Score plot of the first two PCs calculated by the application of PCA on top 500 genes expressed by primary MSCs, HS-5, and HS-27 cell lines following batch effect normalization. **(B)** Normalized gene expression intensities of primary MSCs, HS-5, and HS-27 cell lines within the four different GSE datasets. (*n* MSCs, HS-5, HS-27A = 11, 6, 4).

Supplementary Figure 3 Z-scores related to the expression of all genes included in “angiogenesis” pathway from Hallmark MSigDB (Molecular Signature DataBase) in MSCs, HS-5 and HS-27 cell lines.

Supplementary Figure 4 Evaluation of Fas/FasL expression in primary MSCs, HS-5 and HS-27A cell lines. **(A)** Fas/FasL expression in primary MSCs, HS-5 and HS-27A cell lines in resting condition. **(B)** Fas/FasL expression in primary MSCs, HS-5 and HS-27A cell lines in resting and primed condition.

Supplementary Figure 5 Immunological characterization of HS-5 and HS-27A cell lines. **(A,B)** Relative PBMCs proliferation following 4 days of co-culture with γ-irradiated resting or primed HS-5 **(A)** or HS-27A **(B)**. PBMCs proliferation was calculated on living CD45^+^ cells according to CFSE dilution method by measuring CFSE gMFI and normalized on activated PBMCs cultured in absence of stromal cells. Data are represented as mean ± SEM. **(C)** Representative proliferation of living CFSE^+^CD45^+^ non-activated PBMCs following the co-culture with resting or primed MSCs, HS-5, and HS-27A.
