# Supplementary figures and images for: HS-5 and HS-27A Stromal Cell Lines to Study Bone Marrow Mesenchymal Stromal Cell-Mediated Support to Cancer Development
Source: Front Cell Dev Biol. 2020 Nov 5;8:584232. doi: 10.3389/fcell.2020.584232 (PMC7674674; doi:10.3389/fcell.2020.584232)

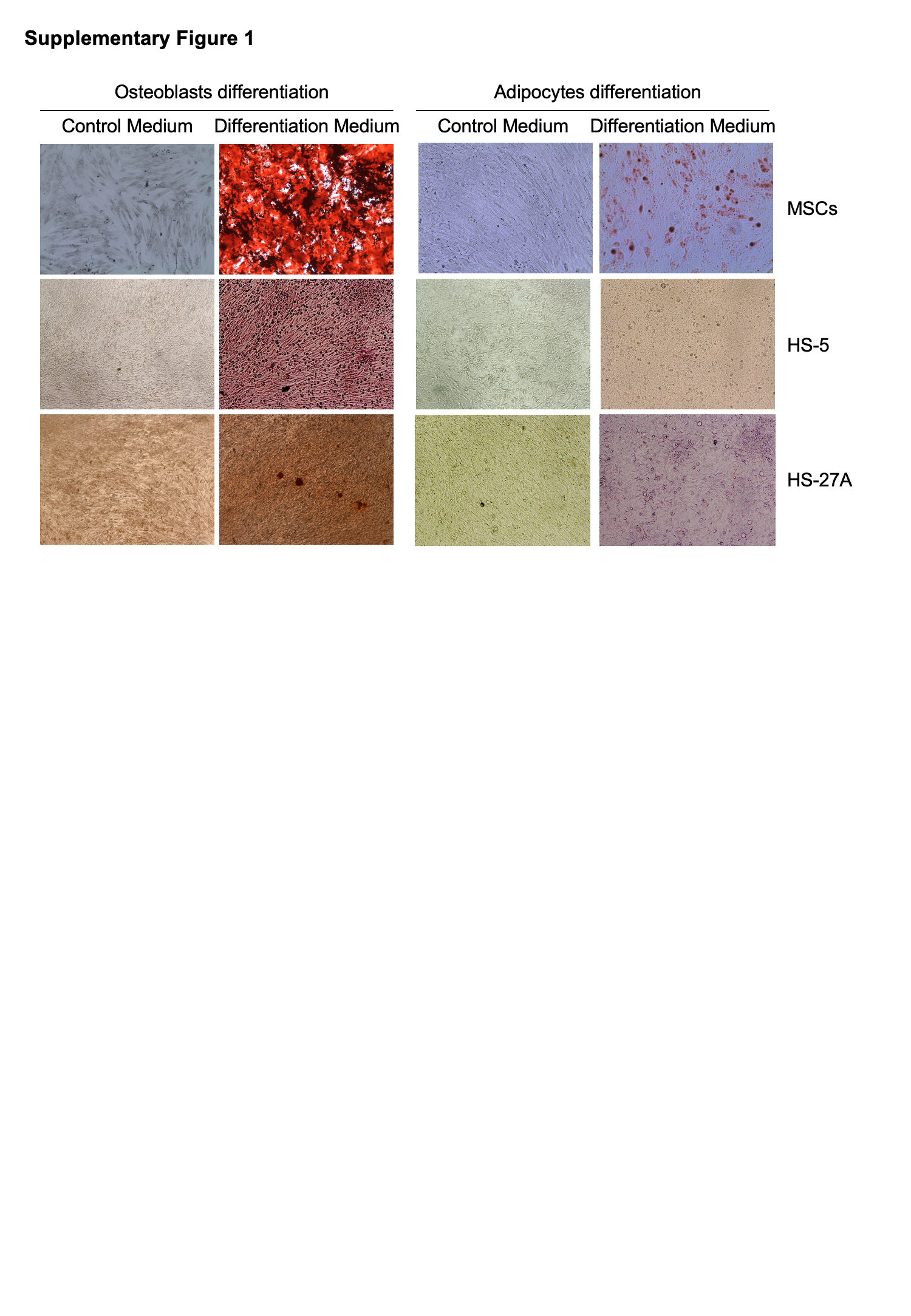

Supplement: Supplementary Figure 1 — Osteoblasts and adipocytes differentiation of primary MSCs and irradiated stromal cell lines HS-5 and HS-27A. [file Image_1.tiff]

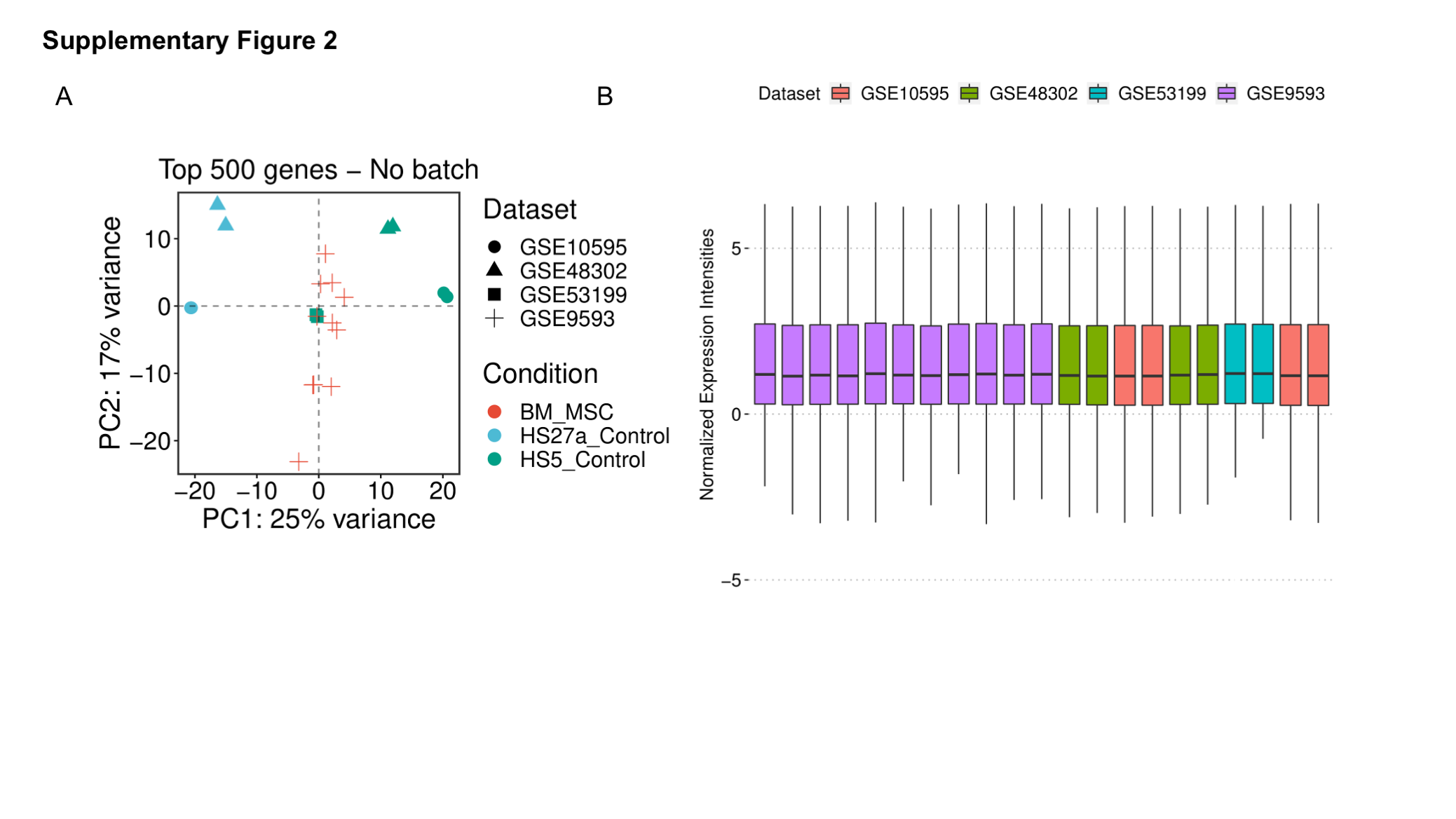

Supplement: Supplementary Figure 2 — Overall gene expression profile of primary MSCs, HS-5, and HS-27A cell lines following batch effect normalization. (A) Score plot of the first two PCs calculated by the application of PCA on top 500 genes expressed by primary MSCs, HS-5, and HS-27 cell lines following batch effect normalization. (B) Normalized gene expression intensities of primary MSCs, HS-5, and HS-27 cell lines within the four different GSE datasets. (n MSCs, HS-5, HS-27A = 11, 6, 4). [file Image_2.tiff]

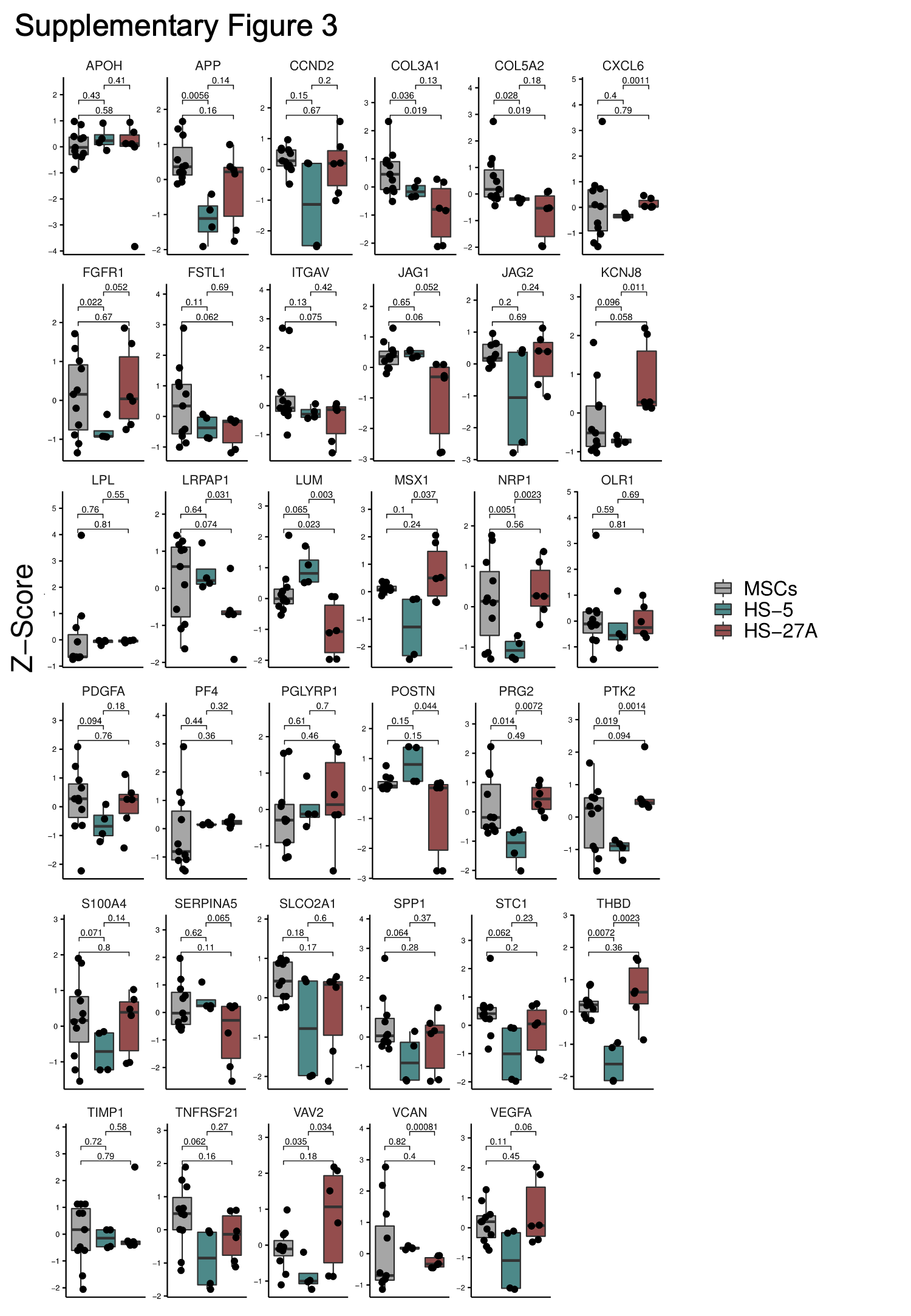

Supplement: Supplementary Figure 3 — Z-scores related to the expression of all genes included in “angiogenesis” pathway from Hallmark MSigDB (Molecular Signature DataBase) in MSCs, HS-5 and HS-27 cell lines. [file Image_3.tiff]

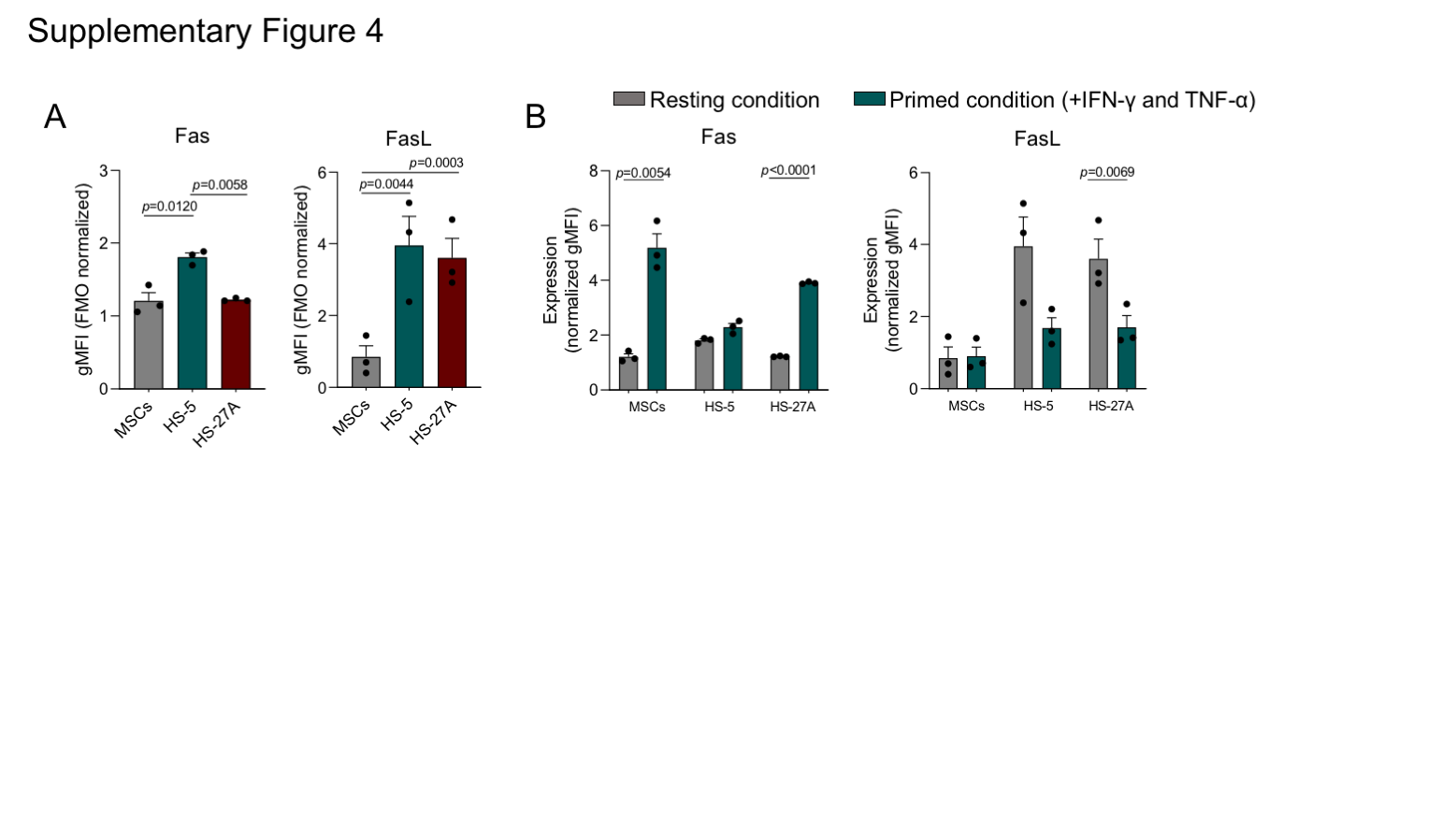

Supplement: Supplementary Figure 4 — Evaluation of Fas/FasL expression in primary MSCs, HS-5 and HS-27A cell lines. (A) Fas/FasL expression in primary MSCs, HS-5 and HS-27A cell lines in resting condition. (B) Fas/FasL expression in primary MSCs, HS-5 and HS-27A cell lines in resting and primed condition. [file Image_4.tiff]

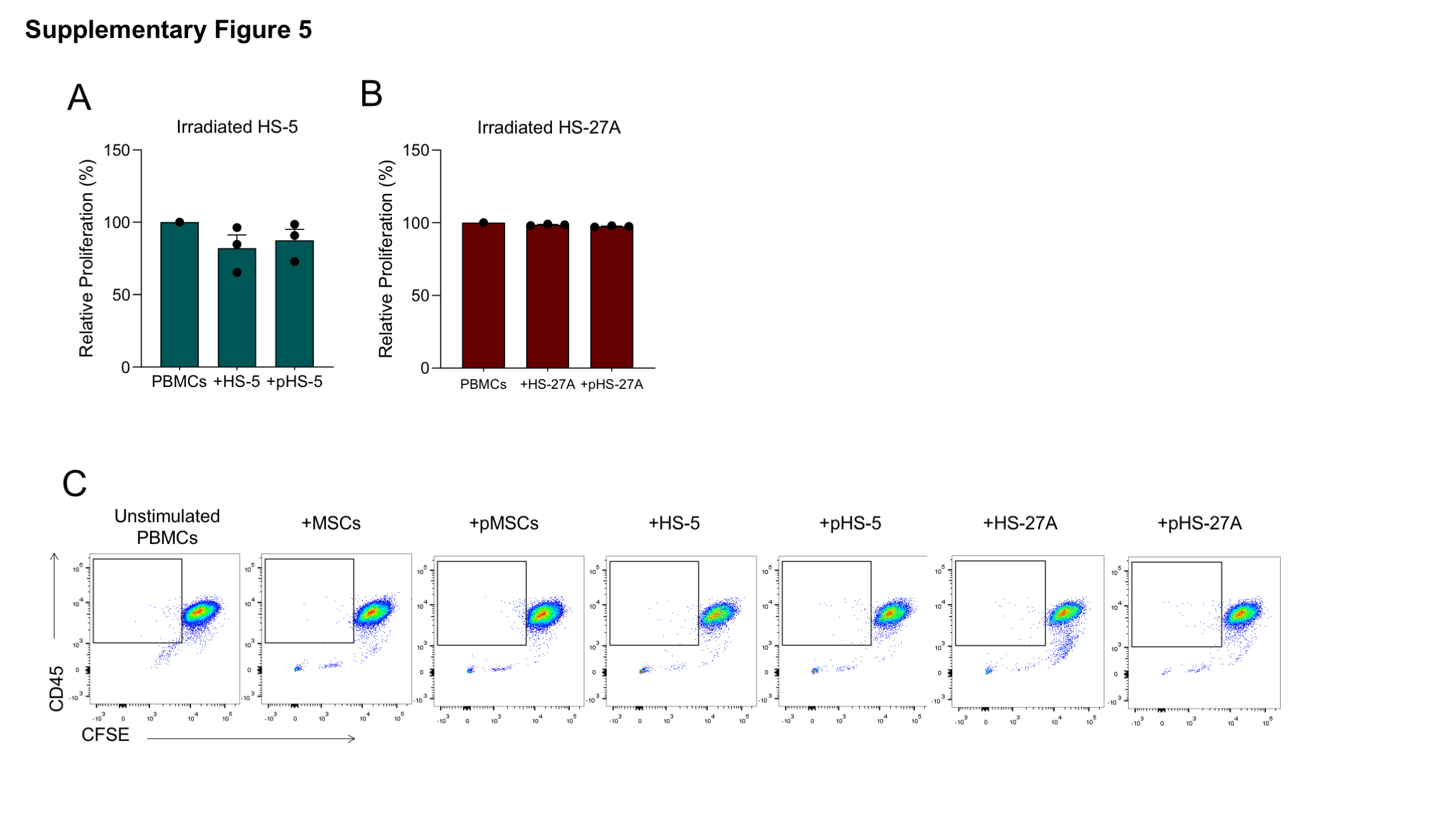

Supplement: Supplementary Figure 5 — Immunological characterization of HS-5 and HS-27A cell lines. (A,B) Relative PBMCs proliferation following 4 days of co-culture with γ-irradiated resting or primed HS-5 (A) or HS-27A (B). PBMCs proliferation was calculated on living CD45+ cells according to CFSE dilution method by measuring CFSE gMFI and normalized on activated PBMCs cultured in absence of stromal cells. Data are represented as mean ± SEM. (C) Representative proliferation of living CFSE+CD45+ non-activated PBMCs following the co-culture with resting or primed MSCs, HS-5, and HS-27A. [file Image_5.tiff]
